# Pupil fluctuations track rapid changes in adrenergic and cholinergic activity in cortex

**DOI:** 10.1038/ncomms13289

**Published:** 2016-11-08

**Authors:** Jacob Reimer, Matthew J McGinley, Yang Liu, Charles Rodenkirch, Qi Wang, David A McCormick, Andreas S Tolias

**Affiliations:** 1 Department of Neuroscience, Baylor College of Medicine, One Baylor Plaza, Houston, Texas 77030, USA; 2 Department of Neuroscience, Yale University, 333 Cedar Street, New Haven, Connecticut 06510, USA; 3 Department of Biomedical Engineering, Columbia University, 1210 Amsterdam Avenue, New York, New York 10027, USA; 4 Department of Electrical and Computer Engineering, Rice University, 6100 Main St, Houston, Texas 77005, USA

## Abstract

Rapid variations in cortical state during wakefulness have a strong influence on neural and behavioural responses and are tightly coupled to changes in pupil size across species. However, the physiological processes linking cortical state and pupil variations are largely unknown. Here we demonstrate that these rapid variations, during both quiet waking and locomotion, are highly correlated with fluctuations in the activity of corticopetal noradrenergic and cholinergic projections. Rapid dilations of the pupil are tightly associated with phasic activity in noradrenergic axons, whereas longer-lasting dilations of the pupil, such as during locomotion, are accompanied by sustained activity in cholinergic axons. Thus, the pupil can be used to sensitively track the activity in multiple neuromodulatory transmitter systems as they control the state of the waking brain.

## Abstract (web summary)

In addition to light intensity, changes in pupil diameter are correlated with mental effort, attention and levels of arousal. Reimer *et al*. report that across behavioural states, fluctuations in pupil diameter are highly correlated with activity of noradrenergic and cholinergic projection neurons.

## Graphical abstract

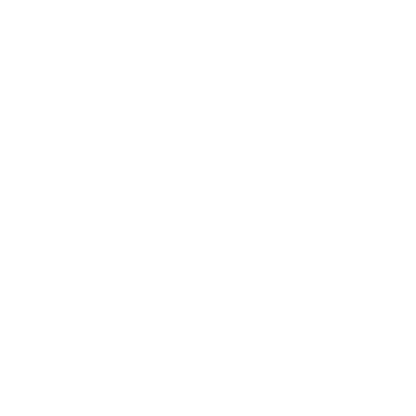

Fifty years of pupillometry in humans and nonhuman primates support the view that in addition to changes in luminance and accommodation, spontaneous fluctuations in pupil diameter track changes in alertness, attention and mental effort^1^,^2^,^3^. In addition, rapid fluctuations in pupil diameter are highly correlated with alterations in electrophysiologically measured brain states, neural responsiveness and behavioural performance^4^,^5^,^6^,^7^. The precise pathways by which these alterations in brain state are coupled to pupil size are unknown. Pupil size has been widely assumed to be a reliable indicator of activity in the locus coeruleus (LC)^8^,^9^ and cortical state is powerfully controlled by the release of acetylcholine (ACh)^10^,^11^,^12^ and norepinephrine (NE)^13^,^14^,^15^.

Here we studied the relationship of activity in cholinergic or noradrenergic axons in the neocortex in relation to spontaneous alterations in arousal (pupil diameter) and locomotion. We find that activity in noradrenergic projections in cortex tracks the phasic changes in pupil diameter better than the cholinergic terminals. On the other hand, cholinergic projections showed more tonic activation when the pupil was dilated for a longer time such as during locomotion.

## Results

### GCaMP6s imaging of cholinergic and noradrenergic projections

**Figure 1:**
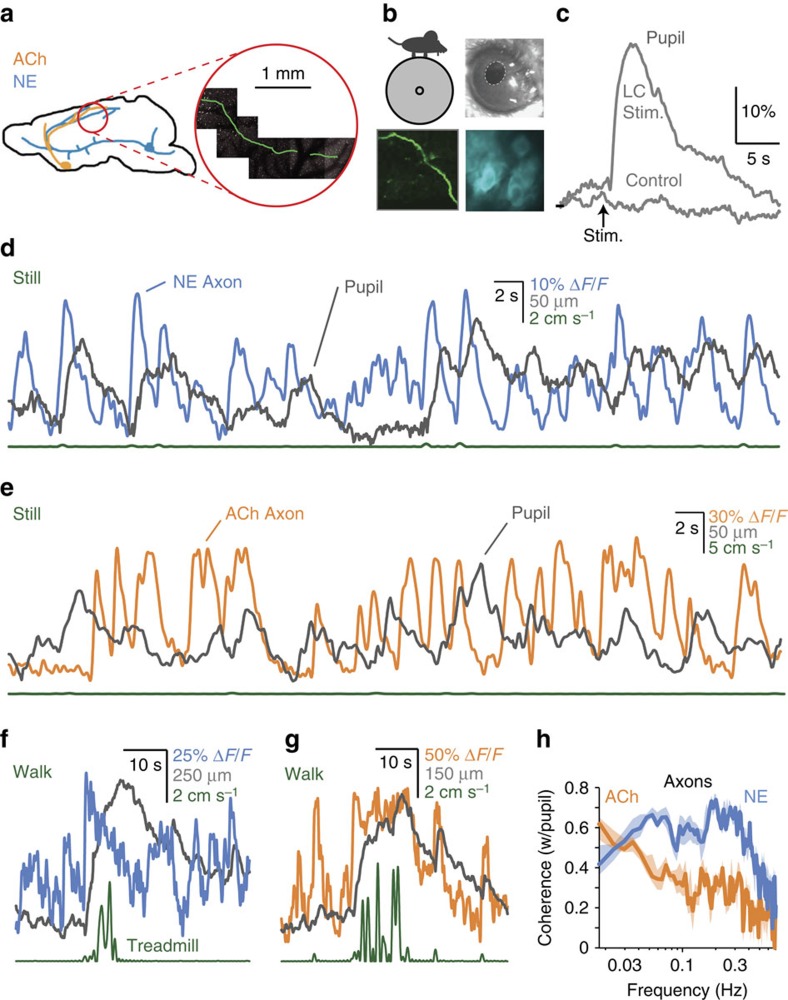
The pupil tracks rapid fluctuations in ACh and NE cortical projections. (**a**, left) *ChAT* projections from BF (orange) and *DBH* projections from LC (blue). (right) A GCaMP6s-expressing axon traversing long distances in layer 1 of V1. (**b**) Simultaneous recording of (clockwise from upper left) treadmill velocity, pupil size, CNiFERs (see text) and axonal calcium activity. (**c**) Stimulation of the LC, but not immediately adjacent tissue, results in a large, rapid dilation of the pupil. (**d**) Activity in NE axons precedes small, rapid pupil dilations during stillness. (**e**) Activity in ACh axons also tracks rapid pupil dilations during stillness, but to a lesser extent. (**f**) At the beginning of walking, strong NE activity occurs along with pupil dilation. (**g**) ACh activity tracks the large, long-lasting dilation of the pupil that occurs around walking. (**h**) NE activity is coherent with fluctuations in the pupil over a broad range of infra-slow frequencies (blue). ACh activity is also coherent with pupil, particularly at the lowest frequencies, such as occur around walking (orange). Error bands represent 68% bootstrap confidence interval.

We used two-photon microscopy to directly measure activity in ACh and NE projections to layer 1 (L1) of mouse cortex while tracking pupillary fluctuations (Fig. 1). To do so, we imaged GCaMP6s fluorescence in axonal projections in primary visual (V1) and auditory (A1) cortex, originating from choline acetyltransferase (*ChAT*)-expressing (cholinergic) neurons in the basal forebrain (*n*=73 recordings from 25 imaging sites in six mice) or dopamine beta-hydroxylase (*DBH*)-expressing (noradrenergic) axons in V1 from neurons in the LC (*n*=66 recordings from 18 sites in three mice; see Methods). Activity in NE and ACh projections in L1 of wakeful mice was highly coherent with pupil fluctuations, particularly at frequencies between 0.03 and 0.4 Hz for noradrenergic axons and <0.03 Hz for cholinergic axons (Fig. 1h and Supplementary Fig. 1C,D; example traces, Fig. 1d–h and Supplementary Figs 2–4). Consistent with this observation, electrical stimulation of the LC, but not adjacent brain areas, resulted in large, rapid dilation of the pupil after a 1.1±0.1 s lag (Fig. 1C; *N*=3 animals). Changes in pupil diameter, mediated by constriction and relaxation of smooth muscles, are well known to be lagged in response to neural activity in the sympathetic and parasympathetic pathways controlling these muscles^16^.

### ACh and NE activity during fluctuations in pupil size

**Figure 2:**
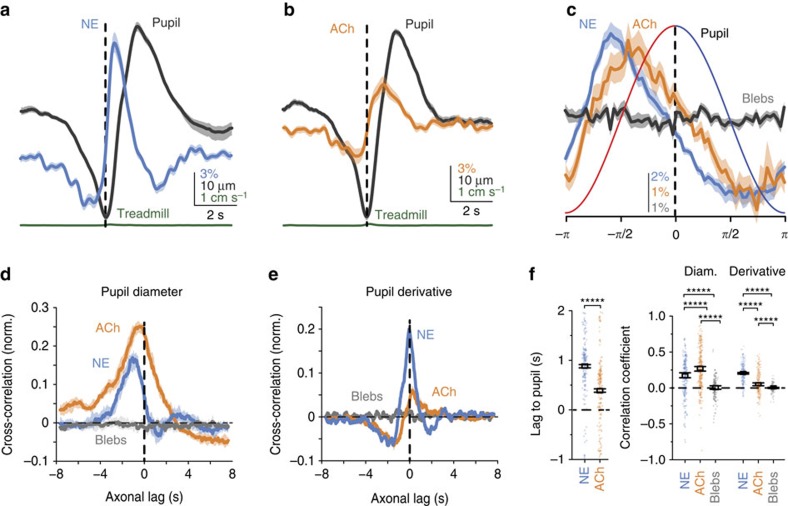
Pupil dilation during stillness is preceded by sequential activation of NE and ACh axons. (**a**,**b**) Mean activity of NE axons (**a**, blue) and ACh axons (**b**, orange) aligned to the onset of dilation. (**c**) NE axons (blue) and ACh axons (orange) aligned to one canonical cycle of dilation and constriction derived from the Hilbert transform (see Methods). No modulation was observed for control auto-fluorescent blebs (grey). (**d**,**e**) Median cross-correlation between ACh, NE or bleb traces and the pupil (**d**) or pupil derivative (**e**). (**f**) The peak in cross-correlation for NE leads ACh with respect to pupil dilation (left). Lag-corrected correlation coefficients between NE, ACh or bleb traces and the pupil diameter or pupil derivative (right). These results suggest that NE activity drives rapid pupil dilations—or is tightly controlled by a separate driver—and that pupil diameter tracks both NE and ACh axonal activity during stillness. Error bands and bars are a 68% bootstrap confidence interval.

During stillness both cholinergic and noradrenergic axonal activity was elevated while the pupil was dilating, and was reduced during constriction. NE activity levels were larger and shorter latency than ACh preceding the peak of a dilation (Fig. 2a–c and Supplementary Figs 2–4). Both ACh and NE activity showed a large, seconds-long peak in cross-correlation with pupil, but only NE activity showed a large peak in cross-correlation to the time derivative of the pupil (Fig. 2d,e). The time of the peak cross-correlation with pupil occurred earlier for NE than for ACh activity (*P*<10^−9^), and both preceded pupil dilation (Fig. 2f, left; NE, lag=0.98±0.04 s, *p*=10^−16^, *n*=75; ACh, lag=0.52±0.13 s, *p*=10^−16^, *n*=140). Both neuromodulators were well correlated with pupil diameter during stillness (non-locomotion) (Fig. 2f, right). ACh activity was more correlated to pupil diameter than was NE, and both neuromodulators were more correlated to pupil diameter than were inactive auto-fluorescent blebs, which were not correlated with pupillary changes (see Methods). The reverse pattern was observed for the correlation to the time derivative of the pupil, which was large for NE and only slightly (but significantly) more correlated for ACh than for blebs (Fig. 2e, right, Supplementary Figs 5–8).

**Figure 3:**
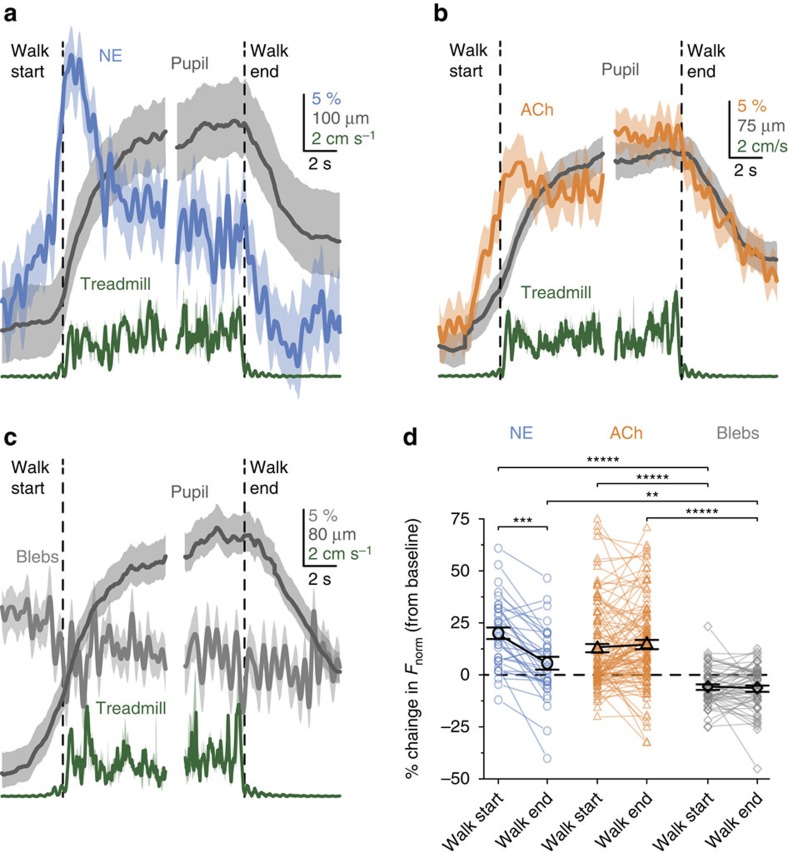
NE and ACh activity display different time courses around walking. (**a**–**c**) Mean calcium trace for NE axons (**a**), ACh axons (**b**) or blebs (**c**), aligned to the start (left) and end (right) of running. Pupil diameter (grey) and treadmill velocity (dark green) are plotted on the same time base. (**d**) A large increase in activity during the first second of walking is apparent in NE and ACh axons, but not blebs. During the last second of walking, NE activity has reduced significantly to near baseline, whereas ACh activity remains high. Error bands and bars are a 68% bootstrap confidence interval.

The activity of both ACh and NE projections increased along with pupil diameter before the onset of walking (Fig. 3). After walking onset, NE activity began to decay whereas ACh activation, and a large pupil diameter, were sustained throughout the walking period in both cortical areas V1 and A1 (Fig. 3a,b, left and Fig. 3d; Supplementary Fig. 8c). The offset of locomotion recapitulated this pattern, with a slow decay of ACh activity that tracked the slow decay of the pupil, and a fast decay in the remaining NE activity (Fig. 3a,b, right). Monitoring of fluorescent blebs showed no walking-related modulation, indicating that the imaging plane was stable during walking (Fig. 3c). Consistent with this pattern, ACh (but not NE) activity was correlated to pupil during walking (Supplementary Fig. 8a). NE activity remained correlated to the pupil derivative during walking, suggesting that small and rapid changes in the pupil diameter during walking reflect NE activity, whereas, the pronounced long-lasting dilation around walking tracks ACh (Supplementary Fig. 8b,c).

### CNiFER imaging of ACh and NE release in V1

To confirm the release of ACh and NE in relation to periods of locomotion, we performed two-photon imaging of HEK-293 cells overexpressing either muscarinic ACh (M1) or α1a NE receptors and a genetically encoded calcium indicator (CNiFERs^17^,^18^) injected into the supragranular layers of V1. These cells change their fluorescence in response to changes in the extracellular concentration of the neurotransmitter for which they express the receptor (for example, Ach and NE), although the response time of these fluorescent indicators is slow (seconds^17^,^18^) and therefore do not permit the examination of rapid changes in transmitter concentration. Bouts of walking were associated with marked increases in the activation of CNiFERs containing either M1-muscarinic or alpha1-noradrenergic receptors (*n*=113 sites in 12 animals), confirming that both ACh and NE were released during the axonal activity around locomotion (Supplementary Fig. 9).

## Discussion

The waking state is associated with rapid variations that can strongly influence neural representations and behavioural responses. Remarkably, these rapid variations in state can be tightly tracked by changes in pupil diameter^4^,^5^,^6^,^7^. Through what neural mechanisms might brain state and pupil diameter be coordinated? The present study provides evidence for activity in cholinergic and noradrenergic projections underlying these rapid variations in cortical state, and provides a mechanistic foundation for the use of pupillometry in awake behaving animals. More generally, our results indicate that ACh and NE are not just regulators of slow changes in wakefulness and arousal, but are recruited on a timescale relevant to moment-to-moment fluctuations that track behavioural events, and that these rapid variations can be partially monitored through pupillometry.

For decades, brain state has been tracked by both changes in electrophysiological parameters^19^,^20^, and by changes in diameter of the pupil^21^,^22^,^23^. Human and animal studies demonstrate that non-luminance and non-accommodation changes in pupil diameter are related to a wide variety of mental and emotional factors, including arousal, attention, stress and cognitive load, and reveal a tight coupling between the state of the central and peripheral nervous systems^1^,^21^,^22^,^23^,^24^,^25^,^26^,^27^,^28^,^29^,^30^,^31^,^32^,^33^,^34^,^35^,^36^,^37^,^38^,^39^,^40^.

At the network level, active behaviours such as locomotion and whisking are associated with a reduction in low-frequency rhythmic cortical activity^6^,^41^,^42^,^43^,^44^,^45^,^46^. Even in the absence of overt movement, increases in pupil diameter (dilation) are associated with increases in cortical activation and suppression of low-frequency rhythms. Likewise, low-frequency cortical activity is enhanced during pupillary constriction, especially below a critical level of pupil diameter. This striking relationship between changes in pupil diameter and cortical network activity, either at the local field potential level^7^ or membrane potential level^5^,^6^ has been observed throughout broad regions of the cortex. Indeed, there is even a strong correlation between pupil diameter and the rate of sharp-wave ripples in the hippocampus^6^, further emphasizing the generality of the relationship between pupil diameter and brain state.

What are the neural pathways that couple brain state and pupil diameter together? A wide variety of neuromodulatory pathways have been implicated in the neural control of brain state (reviewed in refs 47, 48). Two of these are the LC, which provides the source of noradrenergic innervation, and the basal forebrain, which is the source of cholinergic innervation to the cortex^49^. These neurotransmitters can modulate the state of cortical activity through cell type-specific and subcellular mechanisms^5^,^6^,^50^,^51^. Both cholinergic and noradrenergic neurons show graded and transient increases in firing in relation to increased attention to external stimuli, arousal and locomotion^13^,^48^,^52^,^53^. Stimulation of the basal forebrain and brainstem can have many of the same effects as those associated with arousal and locomotion, including increased amplitude and precision of sensory-evoked responses^10^,^11^,^54^. Stimulation of the LC can markedly enhance sensory cortical responses and increase learning induced plasticity^55^. LC neurons have been reported to discharge in close relation to pupil diameter^9^,^13^,^56^. Electrical stimulation in the region of the LC, which may also activate other adjacent non-noradrenergic neurons, results in pupil dilation (Fig. 1). The LC receives synaptic inputs from multiple brain regions, such as the frontal cortex, that may be involved in arousal responses to complex stimuli, including those requiring high level cognition^13^.

What are the unique contributions of cholinergic and noradrenergic pathways to brain state? We, and others^52^,^53^, have observed a tight relationship between movement and basal forebrain cholinergic activity, which is also found when monitoring the activity of brainstem cholinergic nuclei^54^. This raises the question—are the changes in cholinergic activity related simply to arousal or do they represent pre-motor planning, or both? Movement and arousal are intimately interlinked. Here we observed that periods of pupil dilation that were not associated with walking are often associated with increased activity in both cholinergic and noradrenergic fibres in the neocortex, although it is possible that smaller body movements (for example, postural adjustments, whisker movements and so on) may have occurred without locomotion^53^. Interestingly, although we observed increases in arousal and locomotion to be associated with increased activity in both cholinergic and noradrenergic pathways, we found that activity in cholinergic pathways more closely matched locomotion throughout the period of walking, while noradrenergic axon activity followed more closely the moment-to-moment fluctuations in pupil dilation, during both quiet rest and locomotion (Figs 2 and 3). These results suggest that the ascending cholinergic and noradrenergic pathways make unique, but overlapping, contributions to the control of cortical networks. Revealing the precise consequences, at the cellular and circuit level, of increased release of NE and ACh will reveal the role of these neuromodulatory pathways in the ascending control of brain state and whether this control is more closely associated with arousal or movement.

## Methods

### Animals and surgery

All procedures were carried out in accordance with the ethical guidelines of the National Institutes of Health and were approved by the Institutional Animal Care and Use Committee (IACUC) of Baylor College of Medicine, Yale University and Columbia University. In this study, we used a total of 21 mice, including male (*n*=13) and female (*n*=8) mice age 5 weeks to 5 months, and three rats. Twelve animals were injected with CNiFERs (see below), including one animal injected with α1a alone, six animals injected with M1 alone, and five animals injected with both α1a and M1. Several of these mice were *SST-Cre/Ai9* (*n*=6) or *PV-Cre/Ai9* (*n*=2) crosses on a C57Bl/6 background. For imaging of *ChAT* and *DBH* axons, we used six *ChAT-Cre* (Jackson labs strain *B6;129S6-Chattm1(cre)Lowl/J*) and three *DBH-Cre* mice (MMRRC Stock#036778-UCD *B6.FVB(Cg)-Tg(Dbh-cre) KH212Gsat/Mmucd*). Expression of GCaMP6s in cholinergic or adrenergic neurons was achieved either via viral injection (four mice) of floxed AAV-GCaMP6 virus (*AAV1-Syn-FLEX-GCaMP6s*, U Penn Vector Core or *AAV5-CAG-DIO-GCaMP6s*, UNC Vector Core) or reporter expression of GCaMP6s (five mice; *B6;129S6-Gt(ROSA)26Sor*^*tm96(CAG-GCaMP6s)Hze*^/J reporter mouse, Jax strain number #024106).

Viral injections were performed stereotactically through a burr hole under isoflurane anaesthesia. Injections were targeted to the basal forebrain (*ChAT-Cre* mice, *AAV1-Syn-FLEX-GCaMP6s*) by a vertical penetration 4 mm lateral and 0.5 mm posterior of Bregma, and ∼1 ul of virus was injected over a period of 10–15 min at a depth of 4–4.5 mm. Injections targeting the LC (*DBH-Cre* mice, *AAV5-CAG-DIO-GCaMP6s*) were performed similarly at coordinates 0.5 mm just above and below those used in a previous study to target the LC^57^. Injections were performed 4–6 weeks before imaging to allow time for viral expression.

Cranial window surgeries over primary visual cortex were performed as described previously^5^. Briefly, a 3 mm cranial window was opened under isoflurane anaesthesia and sealed with a 3 mm glass coverslip (Warner Instruments) and surgical glue (Vetbond, 3M). In several experiments, the dura was removed before applying the coverslip to increase optical access to the cortex. In two mice used for imaging of *ChAT* axons, the craniotomy was made over primary auditory cortex^6^ instead of V1, and in two other mice, dual craniotomies were made over both primary visual and primary auditory cortices. No differences were observed in effects across the two cortical areas, so results were combined from both brain areas.

### LC stimulation

Sprague-Dawley rats (*n*=3) were anaesthetised with isoflurane. A small craniotomy was performed on the left hemisphere over the LC (stereotaxic coordinates: 3.1–3.7 mm caudal to Lamda, 1.2–1.4 mm lateral to the midline and 5.2–6.2 mm deep from brain surface). A single tungsten microelectrode (impedance: 1–2 MΩ) was slowly advanced into the LC using a hydraulic micropositioner (David Kopf, Tujunga, CA). Placement within the LC was determined based on characteristics of the neural activity, specifically: wide spike waveforms (∼2 ms), low spontaneous firing rates (0.5–4 Hz), and elevated firing rate in response to paw or tail pinch. Post experiment, microelectrode placement in the LC was further confirmed by electrolytic lesion and histological analysis for all animals. With eyelids held using retractors and eyes illuminated at 50 lux, pupils were imaged at 50 Hz while phasic stimulation, consisting of six anode-leading biphasic current pulses (60 μA, 200 μS phases) at 330 Hz, was delivered through the microelectrode every minute. This same recording and stimulation was then repeated with the microelectrode retracted 500 μM as a control. Resulting pupilometry data was analysed using a custom Matlab script to segment the pupil from the iris and calculate its area over time. Pupil area was normalized by dividing by pupil area prior to stimulation and subtracting unity, resulting in per cent increase in size.

### CNiFERs

In several experiments, HEK-293 cells expressing a calcium indicator and either cholinergic or noradrenergic receptors (CNiFERs) were injected in the cortex. CNiFER cells were plated from frozen stocks (gift of D. Kleinfeld) on 6 cm plates in 3 ml of standard media (high glucose DMEM with pyruvate, supplemented with 10% fetal bovine serum, 1 × non-essential amino acids, and 100 U ml^−1^ penicillin/streptomycin; Life Technologies product numbers 11995065, 10437077, 11140050, 15140122). Cells were passaged or used for experiments when they were at or approaching confluence.

To prepare the cells for injection, media was removed and the plate was washed twice with PBS. Cells were dissociated without trypsinization by pipetting and resuspended in 1 ml ACSF (125 mM NaCl, 5 mM KCl, 10 mM Glucose, 10 mM HEPES, 2 mM CaCl2, 2 mM MgSO4). Aggregates were removed by passing the cells through a pipette-tip cell strainer (40 μm) into a 1.5 ml Eppendorf tube. The cells were then pelleted by lightly spinning the tubes for ∼20 s and most of the supernatant was removed, leaving the cells resuspended in a thick slurry (50–100 ul). 0.5 ul of 1 mM Alexa 568 was added to the cells to facilitate visualization during injection under two-photon microscopy, and they were back-filled into a glass pipette with the tip broken to a diameter of ∼30 μm. Using a syringe and rubber tube attached to the back of the pipette, a small amount of slurry was expelled from the tip to eliminate any bubbles and load the front of the pipette tip with cells. Finally, cells were injected in cortex under two-photon guidance ∼300 μm below the pia with a brief pulse of positive pressure (50–200 mbar). After injection, the craniotomy was sealed with a glass coverslip as described above.

### Locomotion and Pupillometry

After surgery, the mouse was allowed to recover on the heating pad and then transferred to the microscope. The animal's head was restrained under the microscope objective, and their body was supported by a treadmill that allowed free movement along a single axis of rotation. Treadmill motion was measured using a rotary optical encoder with a resolution of 8,000 counts/revolution. Running periods were defined as periods of two seconds or more with treadmill speeds greater than 0.5 cm s^−1^ after filtering the treadmill trace at 5 Hz (Hamming). Multiple running periods separated by less than two seconds were lumped together as a single running epoch.

Images of the eye were recorded at 1,280 × 1,024 at 10 Hz (DCC1545M camera, Thorlabs, with TML-HP 1 × Telecentric lens, Edmund Optics). In some experiments, the eye was illuminated with a 720 nm light-emitting diode (ThorLabs), but in most cases, the infrared light transmitted from the pupil during two-photon imaging was sufficient. A moderate level of ambient illumination was maintained either by a grey screen presented on a 7″ liquid crystal display monitor (Lilliput 665GL-70NP/HO/Y monitor; 60 Hz scan rate positioned 10 cm away from the eye, covering ∼88° (azimuth) by 72° (elevation) of the contralateral visual field, or via an ultraviolet light-emitting diode (380 nm±20 nm, full-width at half-maximum). *Post-hoc* pupil segmentation was performed semi-automatically with custom MATLAB software as described previously^5^.

### Imaging

Two-photon imaging was performed with a fast resonant scanning system (ThorLabs) mounted on a Sutter objective manipulator. The imaging frame rate was 30–60 Hz. Excitation was via a Ti–sapphire laser (Chameleon Vision, Coherent) tuned to either 800 nm (CNiFERs) or 920 nm (GCaMP6s) with either a 26 × (0.8 NA, Nikon) or 25 × (1.1 NA, Nikon) objective. Power out of the objective was controlled by calibrated rotations of a half-wave attenuator and depended on the magnification of the scan but was typically 20–40 mW. We used ScanImage (Vidrio) to control the imaging system, and custom Labview software to acquire treadmill activity and pupil movies synchronized with the imaging scans.

### Preprocessing of calcium imaging data

Imaging data was motion corrected^5^ and raster artefacts from bidirectional scanning were removed. Regions of interest containing axons, CNiFERs, or control regions with auto-fluorescent ‘blebs' were segmented by hand with custom MATLAB software. Imaging of GCaMP6s fluorescence in cholinergic or noradrenergic axons was performed for 218 axonal segments and 73 small blebs from the same image planes. Of these, image sessions for 26 axonal segments (and 9 blebs in the same imaging sessions) were found to have significant motion artefacts (mean displacement >1 μm from the position corresponding to the centre of mass of aligned images across the session) and thus were excluded from further analysis. Of the remaining stable recordings, the presence of reliable, low-noise calcium activity was assessed by analysing the power spectrum of the calcium traces. Power above 1 Hz was considered ‘noise,' because it was outside the kinetic range for GCaMP6s (ref. 58), and an operational signal-to-noise ratio (SNR) for each recording was calculated as the log of the ratio of the peak power in the range of 0.05–0.5 Hz divided by the average power in the 1–3 Hz range (see Supplementary Fig. 1A,B).

Recordings from 53/192 axonal segments and 43/64 blebs with an SNR<log(20) were considered to be high noise or inactive (see Supplementary Fig. 1A,B). Low-SNR/inactive axonal segments were excluded from further analysis and the low-SNR/inactive blebs were included as a control for comparison to axonal segment activity. The high-SNR blebs were likely synaptic boutons, but they were excluded from further analysis because their identity could not be confirmed for certain. Of the 139 active/high-SNR axonal segments, 73 were cholinergic axons (51 in V1 and 22 in A1) and 66 were noradrenergic axons in V1. The SNR did not differ between virally (7.8±2.4) or transgenically (8.7±2.0) expressed GCaMP6 (*P*=0.14). All calcium traces were re-sampled at 100 Hz and then low-pass filtered at 10 Hz. Following convention, for example traces data are presented as Δ*F*/*F* (where *F* is median fluorescence over the entire trace). However, *F*_norm_=(*F*−*F*_min_)/(*F*_max_−*F*_min_) is used in all other analyses in order not to over- or under-weight highly active calcium traces. The data processing chain for this and subsequent analysis relied on the DataJoint library for MATLAB ( http://datajoint.github.com/datajoint-matlab/)^59^.

### Analysis and statistics

Statistical comparisons were made using the Mann–Whitney *U*-test for single comparisons or analysis of variance followed by Tukey's *post-hoc* when multiple comparisons were made. Asterisks are used in the figures to indicate statistical significance at the following levels: *P<0.05; **P<0.02; ***P<0.005; ****P<0.002; *****P<0.0001. Error bands and bars for all plots were calculated as a 68% bootstrap confidence interval using the BCa algorithm^60^.

### Phase-binned traces

Locomotion periods and saccades were excluded from analysis, and pupil diameter and axon traces were filtered between 0.1 and 1 Hz. For registration to a standard dilation/constriction cycle, the filtered fluorescence trace at each time point was binned by the Hilbert phase of the filtered (0.1 to 1 Hz) pupil trace (64 bins from –*π* to *π*). In this analysis, the data were restricted to periods of pupil dilation and constriction with durations greater than one second and less than ten seconds, and absolute dilation or constriction rates >10 μm s^−1^. Phase plots were smoothed by averaging adjacent bins and normalized to the mean fluorescence across bins before averaging (Fig. 2).

### Coherence and cross-correlations

Coherence was calculated using the multitaper method with adaptive eigen weighting of the first nine Slepian tapers using custom-written code^60^,^61^,^62^. Coherence phase was shifted by±*k* × 360° (where *k* is an integer), until all phases were between −180 and 180° before averaging across recordings. Cross-correlation functions were calculated after low-pass filtering the calcium traces at 10 Hz, subtracting the mean from each trace and dividing by the product of the standard deviations so that the cross-correlation at zero lag is equal to the correlation coefficient between the signals. The lag at peak cross-correlation was determined in a 3 s window spanning zero lag. Cross-correlations that did not achieve a peak in this lag range, and thus where a lag could not be determined, were excluded from further analysis. Lag-corrected correlation coefficients were determined by shifting the calcium traces by the lag with peak cross-correlation and then calculating Pearson's *r* value (Fig. 2 and Supplementary Figs 1,7 and 8).

### Peri-dilation and peri-constriction traces

Locomotion and saccades were excluded from analysis and periods of dilation and constriction were identified in the pupil trace as described above (epochs with durations greater than one second and <10 s, and absolute dilation or constriction rates greater than 10 μm s^−1^). Axon trace segments ±2 s around dilation or constriction onset were averaged for each axon region of interest (ROI), and this mean peri-onset trace was normalized between zero and one (*F*_norm_) before averaging across all ROIs (Fig. 2 and Supplementary Figs 5 and 6).

### Activity around running onset and offset

Locomotion epochs were identified as described above, and normalized traces around the onset or offset of running for each axon or CNiFER ROI were averaged as described above for dilation and constriction. For axon traces, locomotion periods were only used if they were preceded (onset) or followed (offset) by at least 10 s of quiet wakefulness. For CNiFER PSTHs, locomotion periods were included only if they were preceded (followed) by at least 30 s of quiet wakefulness (Fig. 3 and Supplementary Figs 8 and 9).

### Data availability

The data that support the findings of this study are available from the corresponding authors upon request.

## Additional information

**How to cite this article:** Reimer, J. *et al*. Pupil fluctuations track rapid changes in adrenergic and cholinergic activity in cortex. *Nat. Commun.* **7,** 13289 doi: 10.1038/ncomms13289 (2016).

**Publisher's note:** Springer Nature remains neutral with regard to jurisdictional claims in published maps and institutional affiliations.

## Supplementary Material

Supplementary Information Supplementary Figures 1 - 9
