# Supplementary material for: Pupil fluctuations track rapid changes in adrenergic and cholinergic activity in cortex
Source: Nat Commun. 2016 Nov 8;7:13289. doi: 10.1038/ncomms13289 (PMC5105162; doi:10.1038/ncomms13289)
Supplement: Supplementary Information — Supplementary Figures 1 - 9 [file ncomms13289-s1.pdf]

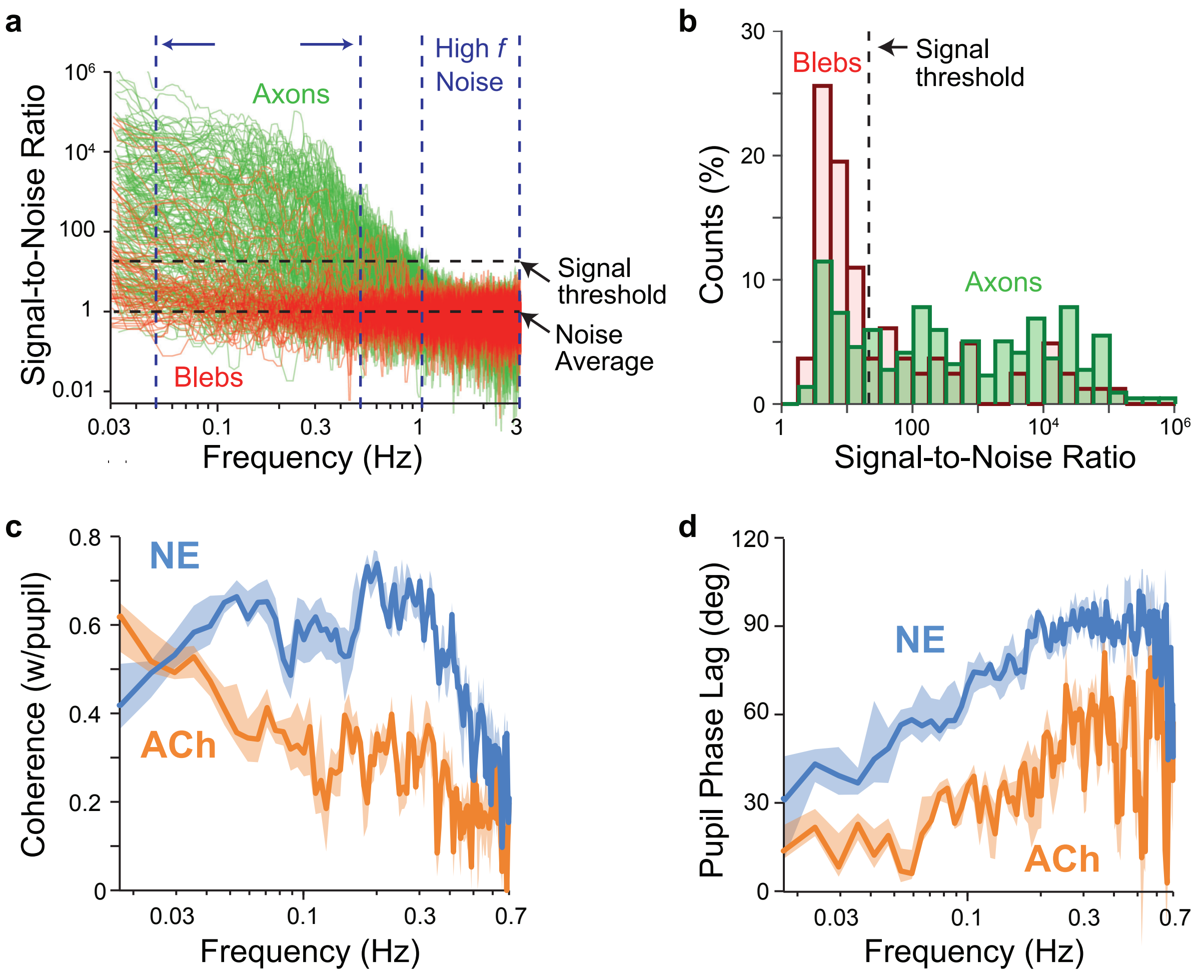

Supplementary Figure 1. Signal-to-noise of neuromodulatory axonal signals and coherence between axonal signals and pupil. **a)** Power spectrum of fluorescent signals from visually identified axonal segments (green traces) and auto-fluorescent blebs (red traces). The spectrum for each trace is normalized to the average of the power in the 1-3 Hz frequency band. Power in the 1-3 Hz frequency band was considered high-frequency 'noise,' because the kinetics of GCaMP6 are not consistent with signal at frequencies above 1 Hz (Chen et al., 2013). Using this operational definition of noise level, axons for which the power crossed a threshold of 20x the noise level in a broad 'signal' frequency band (0.05-0.5 Hz) were considered high signal-to-noise (SNR). **b)** Histogram of the signal-to-noise ratio (peak power in the 0.05-0.5 Hz range divided by the average power in the 1-3 Hz range) for axons and blebs. Vertical dashed line indicates the threshold signal-to-noise ratio (20x) used to define high SNR axons. Note that most blebs had an SNR below this threshold, whereas most axonal signals were above threshold. The high SNR blebs were probably synaptic boutons or axonal segments crossing the imaging plain, but this could not be determined conclusively, so they were excluded from further analysis. **c)** Coherence between pupil and neuromodulators. NE coherence with pupil exhibits a broad peak in the 0.03-0.3 Hz range, whereas ACh coherence was most prominent at frequencies at or below 0.03 Hz (reproduced from Figure 1). **d)** Coherence phase spectra. The pupil exhibits a large, consistent, phase lag with respect to NE activity, which progresses across frequency, consistent with the ~1 second time lag from stimulation of the LC to pupil dilation (see Figure 1C). The pupil also exhibits a consistent, but smaller, phase lag with respect to ACh activity, supporting the view that NE plays more of a driving role in pupil dilation.

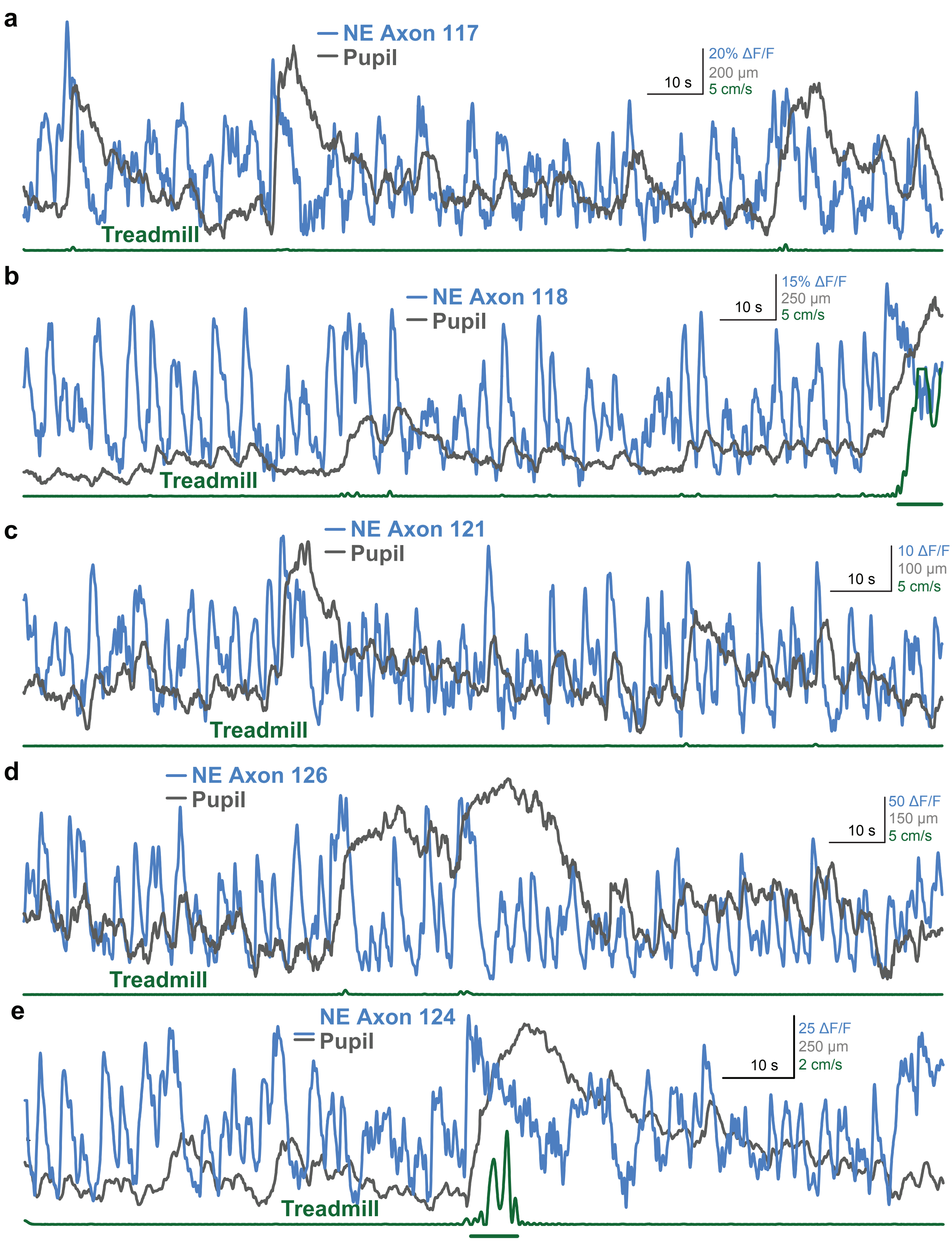

Supplementary Figure 2. Example NE axonal calcium traces (blue) from five recordings in visual cortex (V1). Pupil diameter (grey) and treadmill velocity (dark green) are shown on the same time base. Horizontal green bars indicate walking periods.

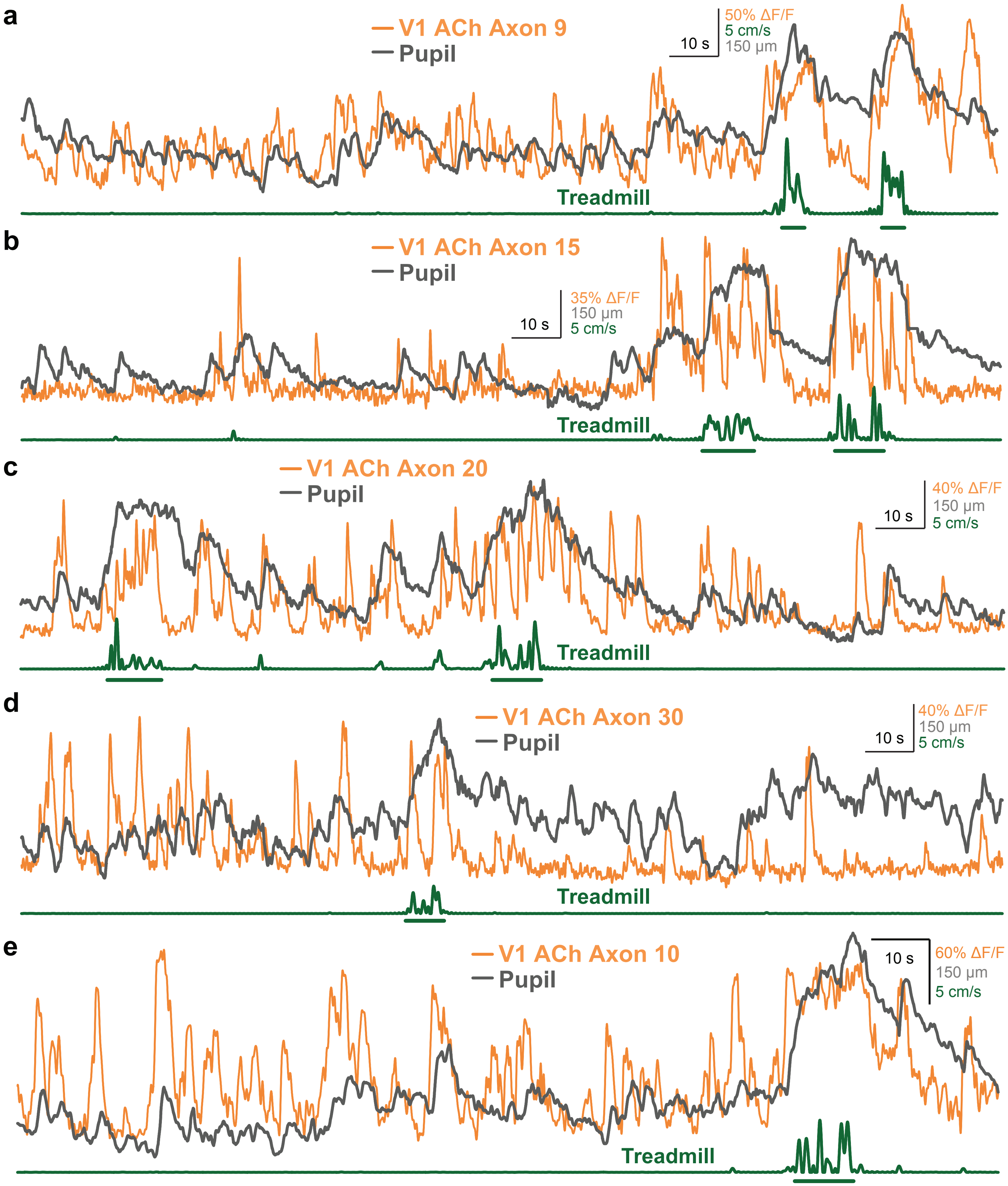

Supplementary Figure 3. Example ACh axonal calcium traces (orange) from five recordings in visual cortex (V1). Pupil diameter (grey) and treadmill velocity (dark green) are shown on the same time base.

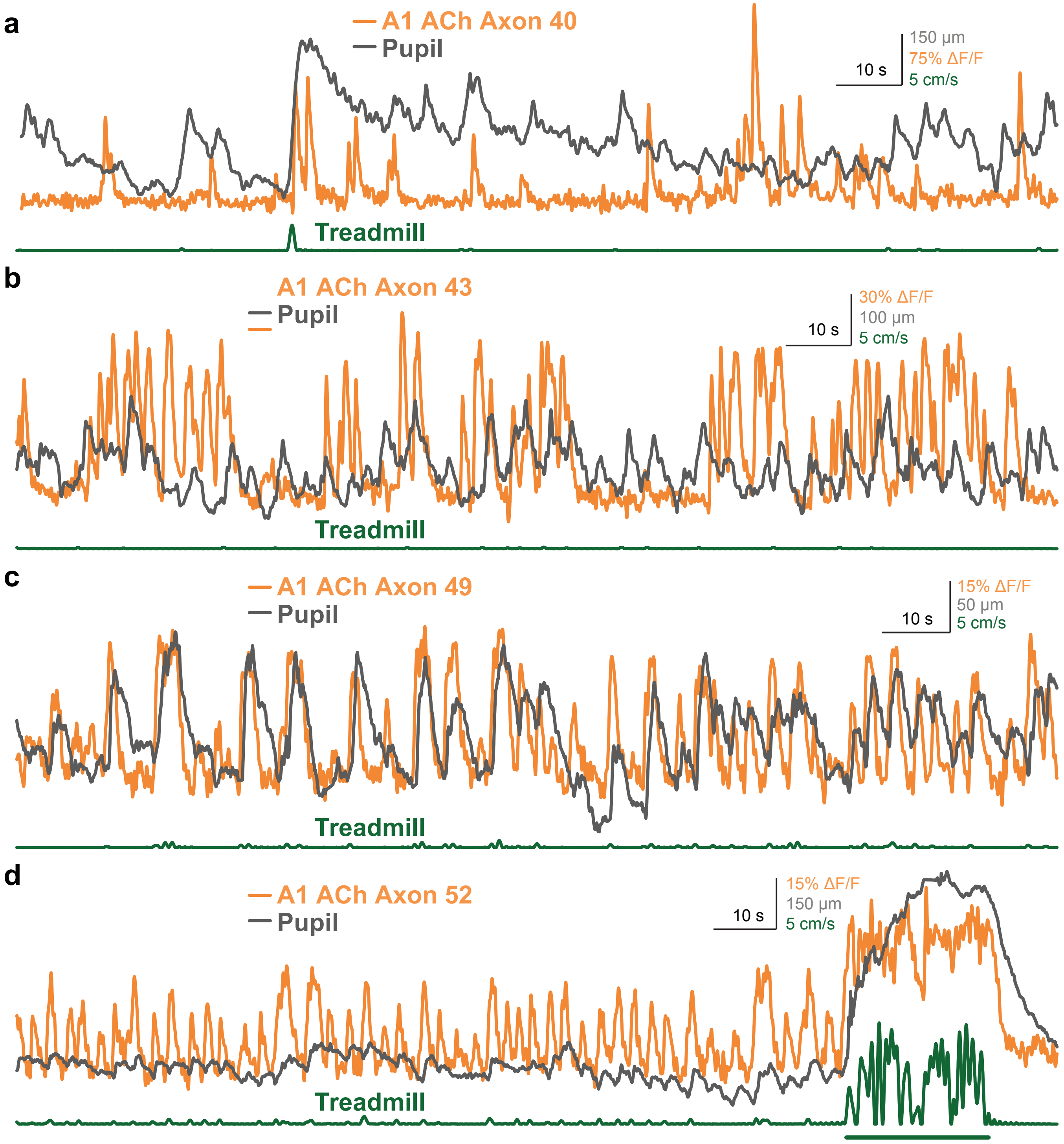

Supplementary Figure 4. Example ACh axonal calcium traces (orange) from four recordings in auditory cortex (A1). Pupil diameter (grey) and treadmill velocity (dark green) are shown on the same time base.

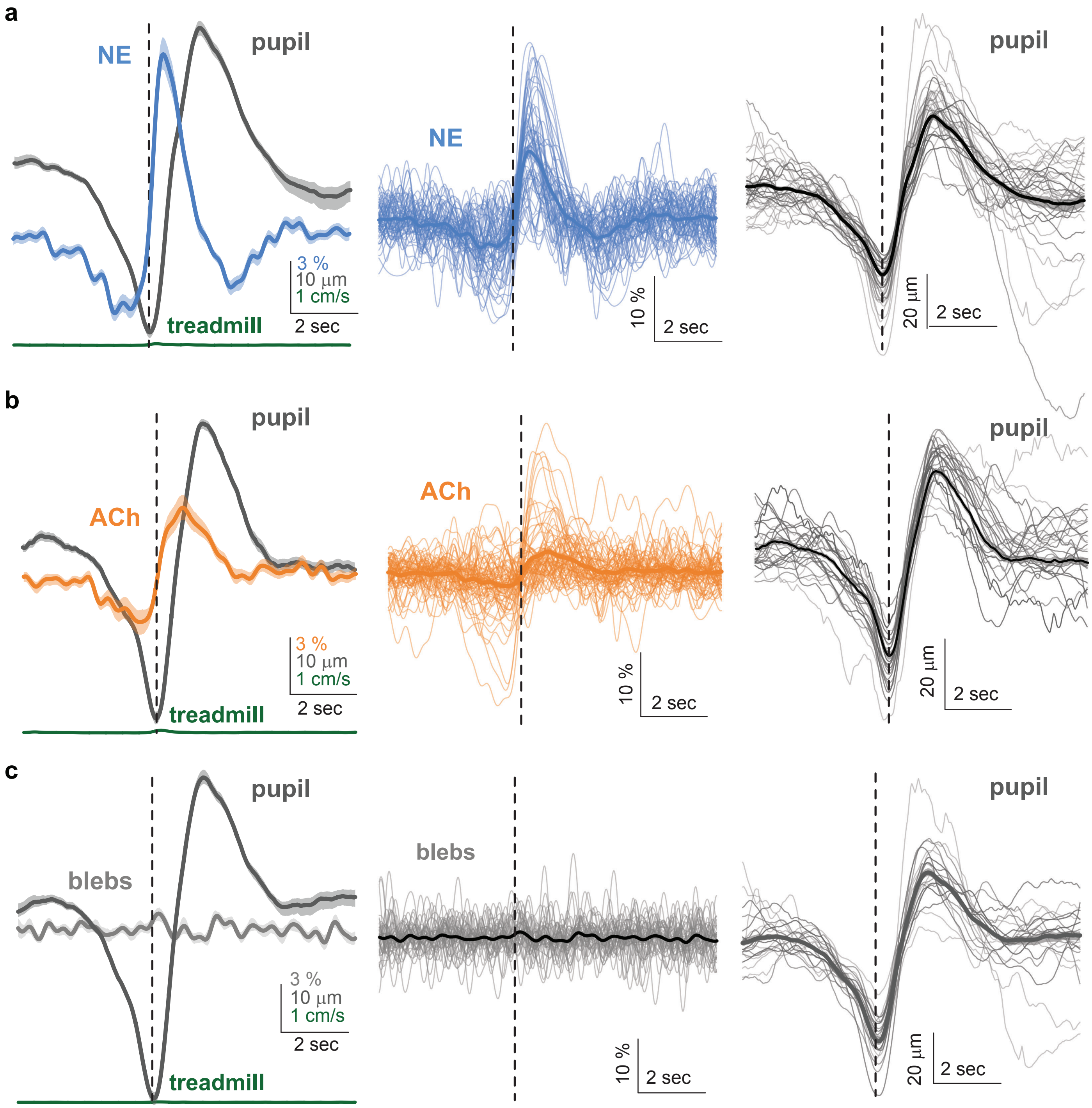

Supplementary Figure 5. Mean neuromodulator activity aligned to dilation onset. **a)** Left: mean NE axonal signal (blue; average across imaging sessions), pupil (grey), and treadmill movement (green) aligned to dilation onset. Middle: superposition of the mean dilation-aligned NE axonal signal from each imaging scan. The mean of these means is superimposed (same trace as in left panel). Right: superposition of the average dilation-aligned pupil diameter from each imaging session. The mean of these means is superimposed (same trace as in left panel). **b)** Dilation-aligned ACh axonal activity (same organization as in panel a). **c)** Dilation-aligned auto-fluorescent bleb activity (same organization as in panels a&b).

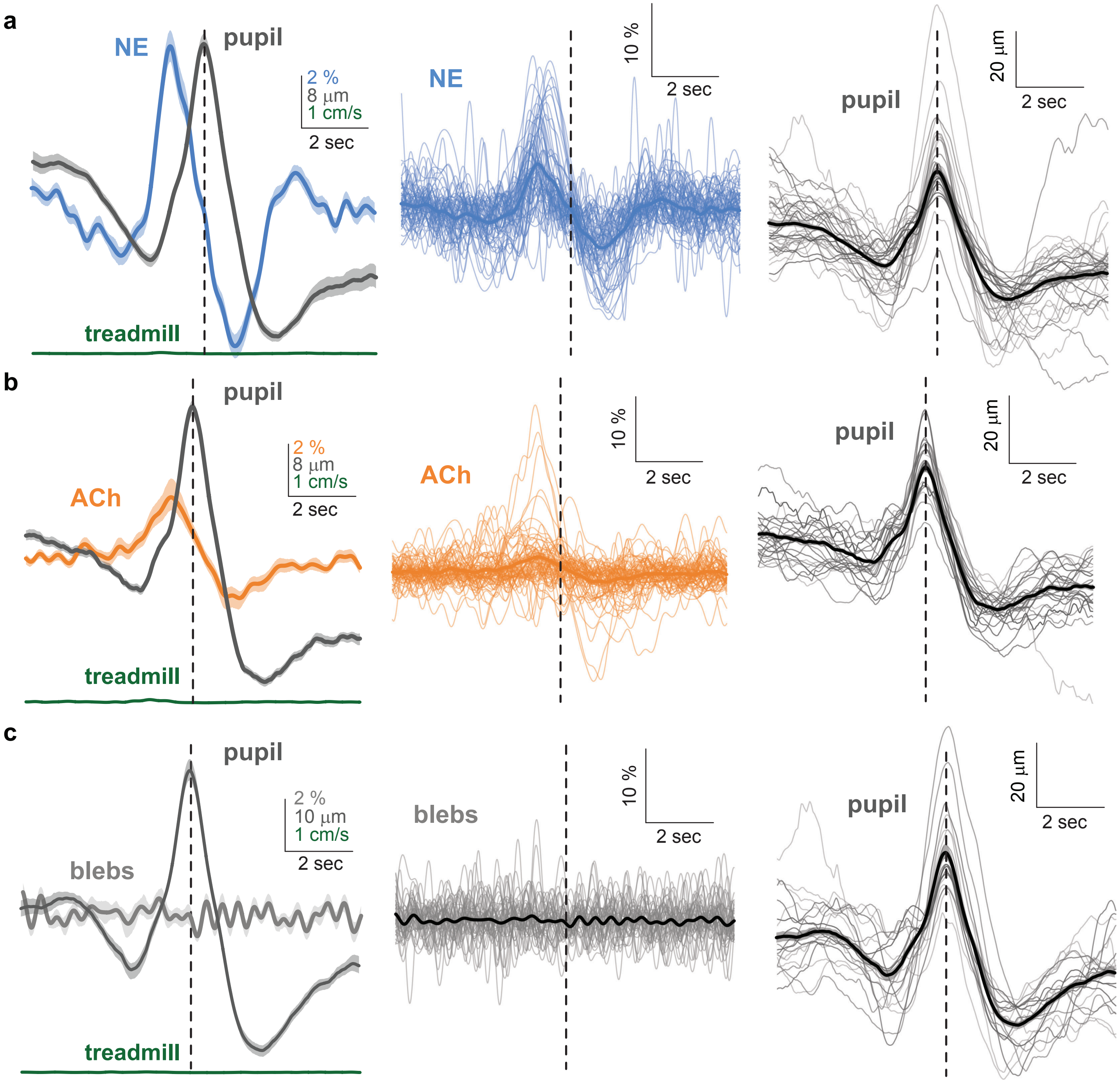

Supplementary Figure 6. Mean neuromodulator activity aligned to constriction onset. **a)** Left: mean NE axonal signal (blue; average across imaging sessions), pupil (grey), and treadmill movement (green) aligned to constriction onset. Middle: superposition of the mean constriction-aligned NE axonal signal from each imaging scan. The mean of these means is superimposed (same trace as in left panel). Right: superposition of the average constriction-aligned pupil diameter from each imaging session. The mean of these means is superimposed (same trace as in left panel).
b) Constriction-aligned ACh axonal activity (same organization as in panel a). **c)** Constriction-aligned auto-fluorescent bleb activity (same organization as in panels a&b).

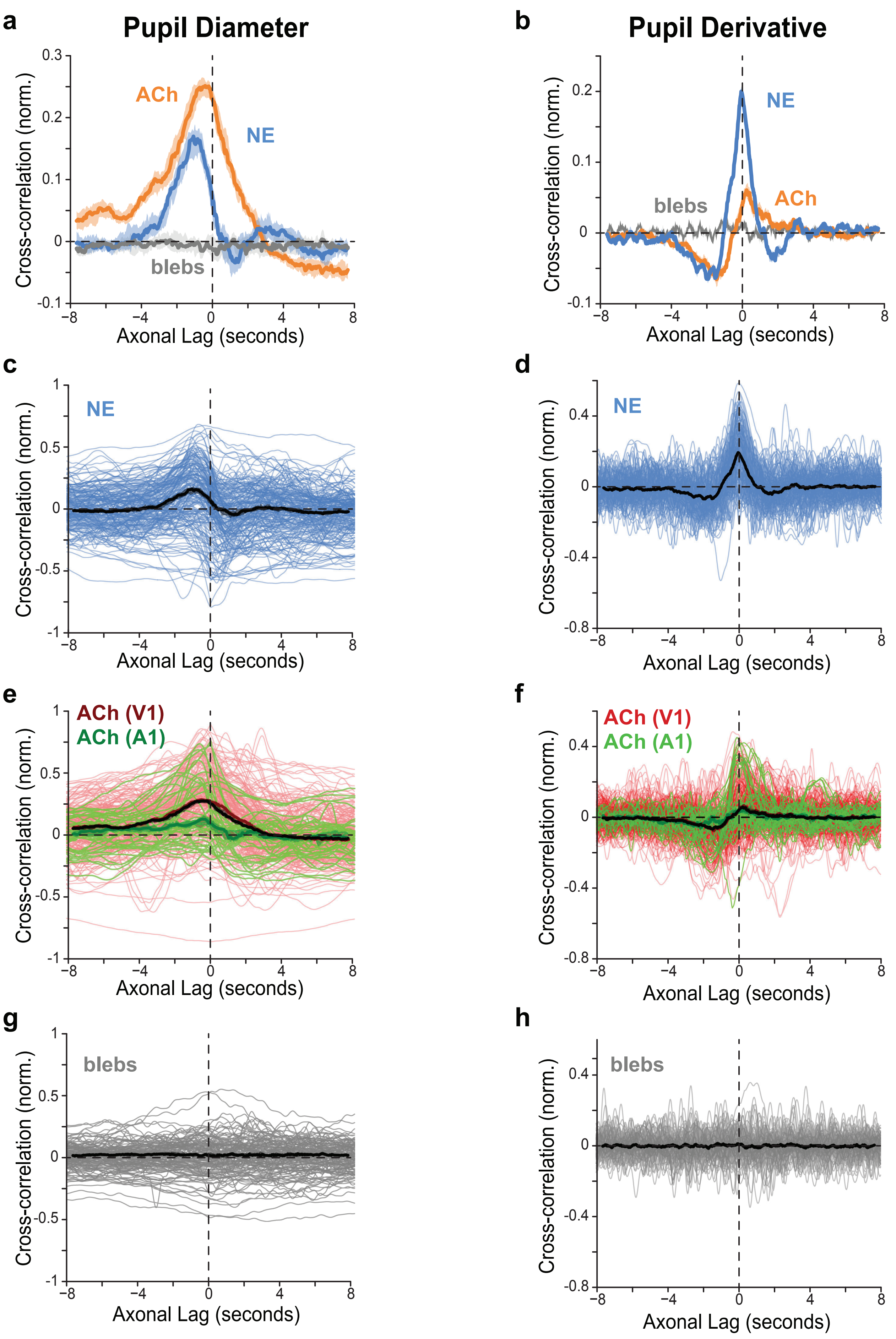
a
Pupil Diameter
b
Pupil Derivative
ACh
NE
blebs
c
d
e
f
g
h
ACh (V1)
ACh (A1)
Cross-correlation (norm.)
Axonal Lag (seconds)

Supplementary Figure 7. Cross-correlation between neuromodulators during stillness. **a-b)** Median cross-correlation between ACh, NE, or bleb traces and the pupil (a) or pupil derivative (b) during periods of stillness (reproduced from Figure 2). **c-d)** Raw NE axonal cross-correlations to pupil (c) and pupil derivative (d) for all still periods from all imaging sessions. Median traces are superimposed in black (same as blue traces in panels a and b). **e-f)** Raw ACh axonal cross-correlations to pupil (e) and pupil derivative (f) for all still periods from all imaging sessions. Traces from V1 are in red and from A1 are in green. Median traces from each brain area are superimposed in darker hue. **g-h)** Raw auto-fluorescent bleb cross-correlations to pupil (c) and pupil derivative (d) for all still periods from all imaging sessions. Median traces are superimposed in black (same as grey traces in panels a and b).

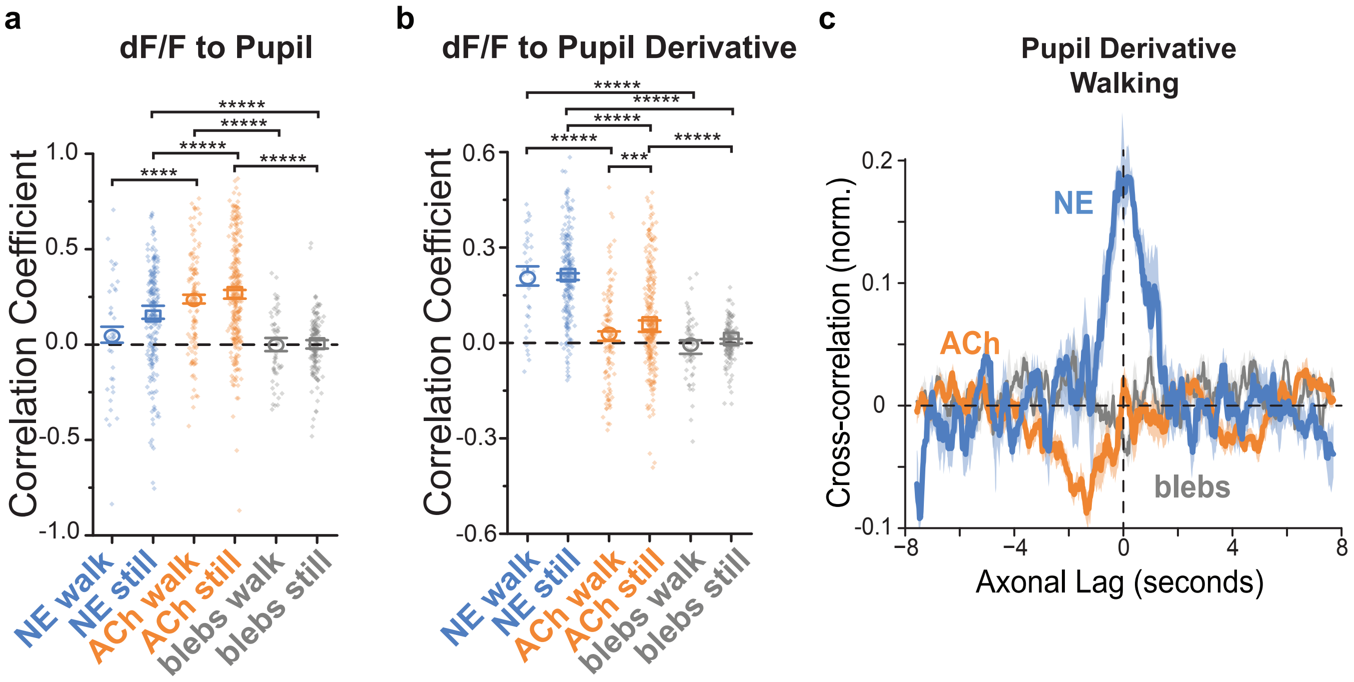

Supplementary Figure 8. Cross-correlations between neuromodulator activities and pupil during walking and stillness. **a)** Lag-corrected correlation coefficients between NE and ACh axonal traces or auto-fluorescent blebs and pupil diameter, during stillness and walking. One-way ANOVA $F_{3,525}$=11.7; group p<2x10-7. **b)** Lag-corrected correlation coefficients between NE and ACh axonal traces or auto-fluorescent blebs and pupil derivative, during stillness and walking. One-way ANOVA F3,525=29.0; group p<1x10-7. The correlation coefficient between ACh activity and pupil was not different between V1 and A1 during stillness or walking ($F_{3,376}$=2.1; Groupwise p=0.1) or for pupil derivative between V1 and A1 during stillness or walking (all pairwise p-values>0.06). However, cholinergic innervation of cortex involves multiple projections, and further exploration is required to understand the diverse roles played by these projections (Zaborszky et al., 2015). **c)** Cross-correlation between NE (blue), ACh (orange), or blebs (grey) and pupil derivative during walking. NE activity remains correlated to the pupil derivative during walking, suggesting the NE may still play a driving role in changing the pupil diameter even during walking, when the pupil is relatively consistently dilated and NE activity is only consistently high at the beginning of walking. *, p<0.05; **, p<0.02; ***, p<0.005; ****, p<0.002; *****, p<0.0001.

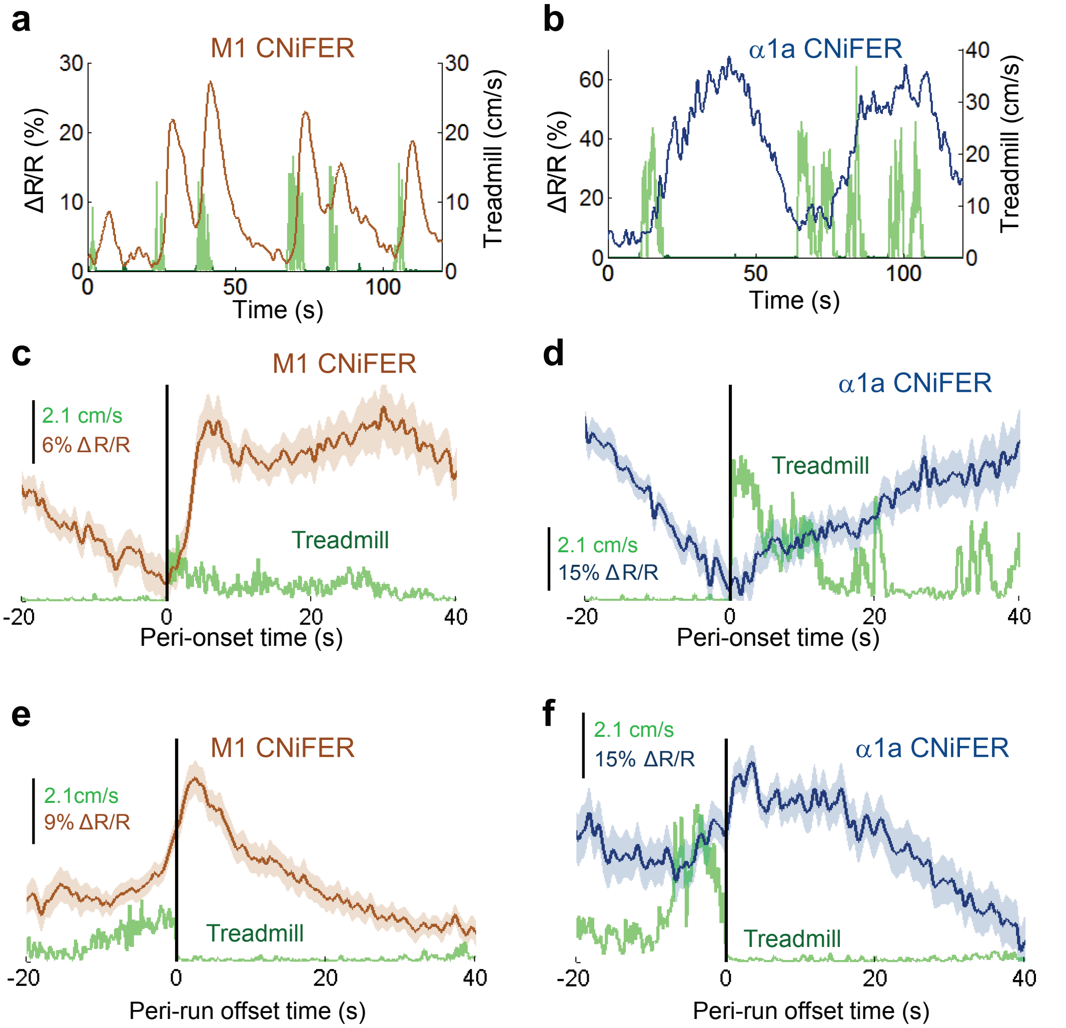

Supplementary Figure 9. M1 and α1a CNiFER activation associated with walking. **a)** Example ACh (M1) CNiFER trace (tan) and treadmill velocity (green) on the same time base. **b)** Example NE (α1a) CNiFER trace (blue) and treadmill velocity (green) on the same time base. **c)** Mean ACh (M1) CNiFER trace aligned to run onset. **d)** Mean NE (α1a) CNiFER trace aligned to run onset. **e)** Mean ACh (M1) CNiFER trace aligned to run offset. **f)** Mean NE (α1a) CNiFER trace aligned to run offset.
